# Clinical foot measurements as a proxy for plantar pressure testing in people with diabetes

**DOI:** 10.1186/s13047-021-00494-4

**Published:** 2021-10-27

**Authors:** Vivienne H. Chuter, Martin J. Spink, Michael David, Sean Lanting, Angela Searle

**Affiliations:** 1 School of Health Sciences, Faculty of Health and Medicine, University of Newcastle, PO Box 127, Ourimbah, NSW 2258 Australia; 2 Priority Research Centre for Physical Activity and Nutrition, University of Newcastle, Callaghan, Australia; 3 School of Medicine, Griffith University, Brisbane, Queensland Australia

**Keywords:** Diabetes, Plantar pressure, Biomechanical

## Abstract

**Background:**

High plantar pressures are associated with increased foot ulcer risk in people with diabetes. Identification of high plantar pressures in people with diabetes is clinically challenging due to time and cost constraints of plantar pressure testing. Factors affecting foot biomechanics, including reduced joint range of motion and foot deformity, are implicated in the development of high plantar pressures and may provide a method to clinically identify those at risk of pressure related complications. The aim of this study was to investigate the contribution of joint range of motion and foot deformity measures on plantar pressures in a community dwelling group with diabetes.

**Methods:**

Barefoot (Tekscan HR Mat™) and in-shoe (Novel Pedar-X®) plantar pressure variables, weight bearing ankle dorsiflexion, hallux range of motion, lesser toe deformities and hallux abductus (HAV) scale were assessed in 136 adults with diabetes (52.2% male; mean age 68.4 years). Multivariate multiple linear regression was used to assess the effect of the four biomechanical factors plus neuropathy and body mass index on plantar pressure variables. Non-parametric bootstrapping was employed to determine the difference in plantar pressure variables for participants with two or more foot biomechanical pathologies compared to those with less than two pathologies.

**Results:**

Almost one third (32%) of the cohort had two or more foot biomechanical pathologies. Participants with two or more foot biomechanical pathologies displayed significant increases in all barefoot plantar pressure regions (except forefoot), compared to those with less than two pathologies. No significant changes were found for the in-shoe plantar pressure variables. The regression model explains between 9.9% (95%CI: 8.4 to 11.4%) and 29.6% (95% CI: 28.2 to 31%), and between 2.5% (1.0 to 4.0%) and 43.8% (95% CI: 42.5–44.9%), of the variance in the barefoot and in-shoe plantar pressure variables respectively.

**Conclusions:**

Participants presenting with two or more factors affecting foot biomechanics displayed higher peak pressures and pressure time integrals in all foot regions compared to those with less than two factors. The tests used in this study could help clinicians detect elevated plantar pressures in people with diabetes and present an opportunity for early preventative interventions.

**Supplementary Information:**

The online version contains supplementary material available at 10.1186/s13047-021-00494-4.

## Background

Identification and subsequent early treatment of clinical factors that contribute to increased risk of foot ulcer and amputation in people with diabetes should be a high priority for primary care clinicians. In 2017 it was estimated that 8.8% of adults worldwide had diabetes, and this is expected to increase to greater than 10% by 2045 [1]. Diabetes-related foot ulcer (DFU) is one of the most common diabetes-related complications, with a lifetime risk of a DFU between 15 and 34% [2, 3], and a global prevalence of DFU of 6.3% [4]. Prevention of initial DFU, and the recurrence of DFU, is crucial as the evidence shows that up to 85% of lower extremity amputations are preceded by DFU [5]. In addition to the high personal costs of DFU and amputation, there are also high associated medical costs, with 20 to 30% of all diabetes-related health care costs spent on foot complications [6, 7].

Peripheral neuropathy is widely recognised as a critical factor in the development of DFU [5], and considerable effort has been dedicated to developing tests that allow clinicians to detect neuropathy with minimal time and cost. These include a 10-g Semmes-Weinsten monofilament, 128-Hz tuning fork, pinprick sensation, ankle reflexes and vibration perception threshold testing using a neurothesiometer [8]. As a result, neuropathy is regularly tested in clinical practice [9]. The International Working Group on the Diabetic Foot (IWGDF) guidelines state that high plantar pressures are also a significant independent risk factor for DFU and should therefore be avoided [10]. Elevated plantar pressures have been associated both prospectively and retrospectively with increased DFU risk in people with diabetes [11–13], with DFU recurrence [14], and may also be predictive in determining specific plantar sites prone to ulceration [12, 15]. However, the cost of plantar pressure testing equipment and the time required for testing and evaluation of data means it is not widely used in general clinical practice [10]. Identification of risk of elevated plantar pressures, by clinic based measures where plantar pressure testing is unavailable, will assist clinicians in determining treatment plans.

Therefore, the aims of this study were to investigate the prevalence of easily measured foot deformities and joint limitations that have been shown to increase plantar pressures [16], in a largely low risk community dwelling group with diabetes. Then, to examine the effect of these factors, plus other factors also associated with elevated plantar pressures (peripheral neuropathy and body mass index [17, 18]) on barefoot and in-shoe plantar pressures. Screening for these factors, individually and in combination, where plantar pressure testing is not available, may provide an early indication of increased ulcer risk in people with diabetes. Early referral of suspected higher risk patients for plantar pressure testing, or initiation of conservative therapies designed to reduce plantar pressures may help preserve functional limbs in this population.

## Methods

### Participants

Participants were recruited from the University of Newcastle Podiatry Clinic at Wyong Hospital, NSW Australia and from newspaper advertisements in local newspapers, between June 2016 and October 2017. Inclusion criteria were adults, 18 years of age and over, ability to speak and read basic English, and a diagnosis of either type 1 or type 2 diabetes only. Exclusion criteria were existing DFU affecting plantar pressure measurement, any previous lower limb amputation, any surgery to the foot or lower limb involving fixation of a joint, any neurological condition that may affect the lower limb other than loss of sensation due to diabetes, inability to walk 8 m unaided, or current pregnancy. Ethics approval was granted by the University of Newcastle Human Research Ethics Committee and written informed consent was obtained from all participants.

### Procedures

All data were collected at one testing session at the University of Newcastle Podiatry Clinic, Wyong Hospital, NSW, Australia. Testing was conducted on the participants’ dominant leg only, to maintain the independence of data [19]. Dominance was determined by asking the participant which foot they would kick a football with. Details of chronic medical conditions and medications, glycated haemoglobin, and duration of diabetes were obtained by self-report and from medical history supplied by the participant’s general practitioner. Neuropathy status was assessed using a monofilament which is a regularly used and reliable test for measuring loss of protective foot sensation (LOPS) [8]. Four points on the plantar surface of the dominant foot (1st, 3rd and 5th metatarsal heads and the distal hallux) were tested with a 10-g Semmes-Weinsten monofilament. An abnormal test was noted and the participant recorded as having neuropathy if they failed to identify the monofilament at one or more test sites [8].

Biomechanical factors were assessed using standard clinic based tests and included weight bearing ankle joint range of motion, hallux abductus valgus (HAV) scale, hallux range of motion, and the presence of lesser toe deformities. Weight bearing ankle dorsiflexion was measured in degrees using a digital inclinometer during a Lunge test with the knee extended. This test has shown excellent intra (ICC = 0.83–0.85, 95%CI: 0.67–0.93) and interrater tester (ICC = 0.88, 85%CI: 0.77–0.94) reliability in a population with diabetes [20]. Participants were recorded as having an ankle dorsiflexion restriction if their weight bearing ankle dorsiflexion was < 30 degrees. This value has been shown to be indicative of an ankle restriction and is associated with increased plantar pressures in older people with diabetes [21]. HAV was assessed by means of the Manchester scale, which uses a set of standardised photographs, with scores ranging from 1 indicating no deformity up to a score of 4 indicating severe deformity [22]. Presence of HAV was defined as a score > 2 for this analysis. Hallux range of motion was assessed in a non-weight bearing position, with a force applied by the examiner to maximally extend the hallux. The degree of dorsiflexion was measured using a goniometer, and the commonly used figure of less than 65 degrees was used to indicate hallux limitus [23]. A visual inspection was conducted to identify lesser toe deformities which included hammer, mallet and claw toes. Toe deformity for this analysis was defined as the presence of any of these deformities on any lesser toe.

Foot pressure testing was conducted barefoot and in-shoe. The Novel Pedar-X® system, (Novel GmbH, Munich, Germany) was used to measure in-shoe plantar pressures, and has been shown to be a reliable and valid measurement system [24]. Participants walked along a flat 12 metre walkway at their normal walking speed wearing an appropriately sized standardised shoe (New Balance® 624), with the insole placed between the sock and the shoe. A minimum of two walking trials was required to capture 12 midgait dominant foot footsteps [24]. Barefoot plantar pressures were collected using the Tekscan HR Mat™ Pressure Measurement System (Tekscan Inc., South Boston, USA) using a 2-step protocol which has been shown to collect reliable pressure data, and an average of four successful trials was used for data analysis [25]. The foot was divided into 5 masks for assessment of both in-shoe and barefoot pressures: rearfoot, midfoot, forefoot, hallux and lateral toes (Additional file 1). Percentage masks were applied for in-shoe footprints where the rearfoot occupied 28% of the total foot length, the midfoot 22%, and the forefoot 30%. Of the remaining 20% of the foot length, the hallux occupied the medial 35% and the lateral toes the lateral 65% [26]. To evaluate the barefoot pressures, a method similar to that used in previous studies to examine plantar loading in older people was applied [27], with the only change being a consolidation of three metatarsophalangeal joint regions into one forefoot region. Pressure time integral (PTI) and peak pressure (PP) values are reported.

### Statistical analysis

Statistical analyses were conducted using Stata 16.0 (StataCorp, College Station, Texas, USA). Descriptive data were summarised as counts (percentages) for categorical variables and means and standard deviations for continuous variables. Due to the association between the 10 pressure outcome variables, multivariate multiple linear regression was used to assess the effect of the four biomechanical factors, plus neuropathy and BMI on plantar pressure variables for both barefoot pressure and in-shoe pressure scenarios. As all models included the same factors, the R-squared statistic was used to assess relative goodness of fit. In addition, appropriate visual model diagnostics (Additional file 2) were checked such as normality of residuals and heteroscedasticity. A measure of the total number of foot biomechanical pathologies was calculated by summing the dichotomous variables of presence weight bearing ankle dorsiflexion restriction, presence of hallux limitus, presence of HAV deformity and presence of any lesser toe deformity. Non-parametric bootstrapping with 1000 iterations was employed to calculate point and interval estimates to determine the percentage change in plantar pressure variables for participants with equal or greater than 2 foot biomechanical pathologies compared to those with less than two pathologies. A two-sided *p* < 0.05 was considered statistically significant.

## Results

One hundred and thirty-six people with diabetes were recruited for the trial (Table 1). The majority of participants had a diagnosis of Type 2 diabetes (90.4%) and were predominantly a low risk cohort based on the low reported levels of diabetes-related complications including a history of DFU (3.7%), retinopathy (2.9%), nephropathy (2.2%) or LOPS with foot deformity (11.8%) [28]. The prevalence of foot biomechanical pathologies was high, with 62% (*n* = 84) of the group having at least one of the assessed foot biomechanical pathologies. A weight bearing ankle dorsiflexion limitation (31%) was the most commonly recorded problem, followed by hallux limitus (28%) and toe deformities (21%) (Table 1). While almost one third (32%) of the cohort had two or more pathologies, none of the participants had all four of the investigated biomechanical foot pathologies.

**Table 1:** Characteristics of the study population

	Total population (***n*** = 136)
Age (years)	68.4 (11.5)
Male (n (%))	71 (52.2%)
BMI (kg/m^2^)	32.7 (6.3)
Type 2 diabetes (n %)	123 (90.4%)
Duration of diabetes (years)	14.6 (11.1)
HbA1c (*n* = 104)	7.2 (1.2)
Insulin therapy alone or combination (n (%))	35 (25.7%)
Oral hypoglycaemics alone or in combination with insulin (n (%))	100 (73.5%)
Diet-controlled diabetes (n (%))	18 (13.2%)
Hypertension (n (%))	98 (72.1%)
Cardiovascular disease (n (%))	36 (26.5%)
History of DFU (n (%))	5 (3.7%)
Monofilament neuropathy (LOPS) (n (%))	43 (31.6%)
LOPS with foot deformity (n (%))	16 (11.8%)
Lunge equinus (n (%))	42 (31%)
Hallux limitus (n (%))	38 (28%)
Hallux abductus valgus score > 2 (n (%))	27 (20%)
Toe deformities > = 1 (n (%))	29 (21%)
Barefoot forefoot peak pressure (kPa)	687.6 (291.5)
In-shoe forefoot peak pressure (kPa)	234.1 (52.1)

Values are mean (standard deviation) unless stated otherwise stated; *BMI* body mass index, *HbA1c* glycated haemoglobin

**Fig. 1:**
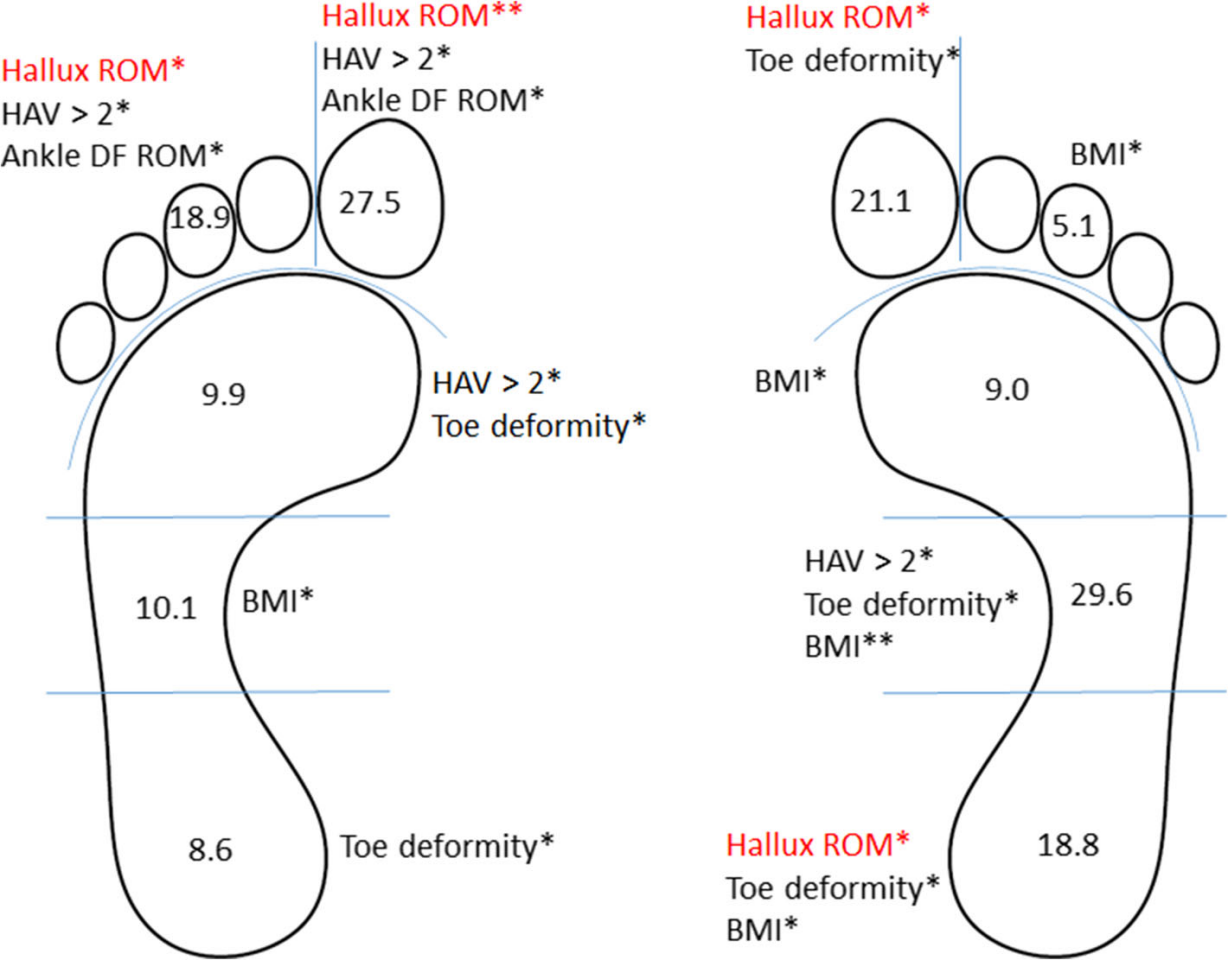
Determinants of barefoot peak plantar pressure regions (left) and pressure time integral regions (right). Values in plantar pressure regions (rearfoot, midfoot, forefoot, lateral toes and hallux) are R-square values and individual predictors significantly associated with each region are listed. Factors in red signify a decrease in plantar pressure. DF, dorsiflexion; *, *p* < 0.05; **, *p* < 0.001; #, no significant factors; BMI, body mass index; HAV, hallux abducto valgus; ROM, range of motion

**Fig. 2:**
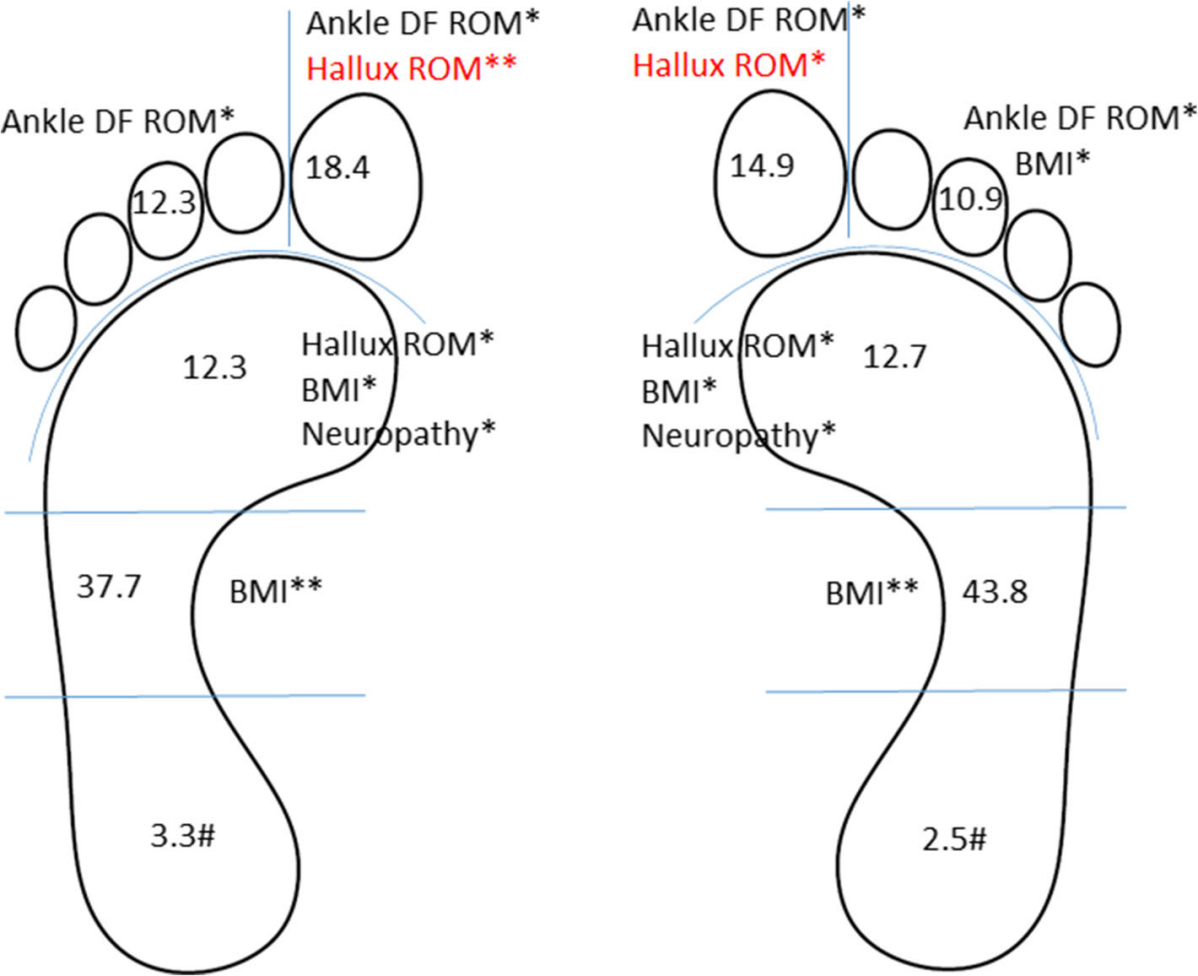
Determinants of in-shoe peak plantar pressure regions (left) and pressure time integral regions (right). Values in plantar pressure regions (rearfoot, midfoot, forefoot, lateral toes and hallux) are R-square values and individual predictors significantly associated with each region are listed. Factors in red signify a decrease in plantar pressure. DF, dorsiflexion; *, *p* < 0.05; **, *p* < 0.001; #, no significant factors; BMI, body mass index; HAV, hallux abducto valgus; ROM, range of motion

The results of the multivariate multiple linear regression, used to identify which of the four biomechanical factors plus neuropathy and BMI contributed to the plantar pressure changes in different foot regions are shown in Figs. 1 and 2. Model diagnostics indicated the results of this modelling to be valid. The model explains between 9.9% (95%CI: 8.4 to 11.4%) and 29.6% (95% CI: 28.2 to 31%) of the variance in the barefoot pressure variables (Fig. 1), and between 2.5% (1.0 to 4.0%) and 43.8% (95% CI: 42.5–44.9%) of the variance in the in-shoe plantar pressure variables (Fig. 2). Individual predictors that were significantly associated with increased or decreased barefoot and in-shoe plantar pressures are illustrated in Figs. 1 and 2 respectively.

When participants were grouped into those with two or more foot biomechanical pathologies and those with less than two pathologies, all but two of the barefoot point estimates (forefoot PP and forefoot PTI), which illustrate the percentage change in plantar pressure variables, were statistically significant (Table 2). The largest increase was seen for toe PTI which was estimated to be 57% (95%CI: 13.4 to 119.3%). No significant changes were found for the in-shoe plantar pressure variables (Table 2).

**Table 2:** Mean (SD) and difference in plantar pressure variables for participants with greater than two foot biomechanical pathologies compared to less than two

	Barefoot pressure variables			In-shoe pressure variables		
	> = 2 ft biomechanical pathologies (***n*** = 43)	< 2 ft biomechanical pathologies (***n*** = 93)	Difference (%) 95% CI	> = 2 ft biomechanical pathologies (***n*** = 43)	< 2 ft biomechanical pathologies (***n*** = 93)	Difference (%) 95% CI
**Peak pressure (kPa)**						
**Rearfoot**	466.8 (238.9)	382.4 (141.1)	22.1^a^ (3.6 to 44.7)	222.0 (60.8)	221.6 (54.3)	0.2 (−9.2 to 10.1)
**Midfoot**	154.4 (134.5)	110.2 (62.3)	40.0^a^ (7.1 to 82.6)	96.8 (27.1)	91.9 (27.9)	5.3 (−5.2 to 16.3)
**Forefoot**	758.3 (277.8)	654.9 (293.4)	15.8 (−1.8 to 32.8)	229.7 (45.2)	236.2 (55.1)	−2.7 (−10.2 to 4.5)
**Toe**	277.9 (158.8)	208.6 (105.3)	33.2^a^ (8.6 to 63.4)	125.1 (50.9)	121.2 (49.5)	3.3 (−11.1 to 20.3)
**Hallux**	457.1 (343.5)	338.3 (179.8)	35.1^a^ (5.4 to 69.7)	187.8 (77.1)	188.0 (68.8)	−0.1 (− 13.6 to 14.7)
**Pressure time integrals (kPa**^**a**^**s)**						
**Rearfoot**	81.6 (30.5)	69.2 (28.3)	17.9^a^ (2.9 to 35.8)	78.4 (38.0)	75.3 (33.9)	4.0 (−11.0 to 22.9)
**Midfoot**	34.7 (24.6)	25.4 (15.1)	36.6^a^ (6.9 to 72.6)	40.8 (14.8)	37.3 (13.2)	9.2 (−3.9 to 24.1)
**Forefoot**	88.2 (30.3)	84.8 (35.2)	4.0 (−7.4 to 18.0)	80.9 (22.2)	83.7 (25.4)	−3.4 (−12.4 to 6.8)
**Toe**	55.9 (58.2)	35.6 (24.8)	57.0^a^ (13.4 to 119.3)	37.8 (15.6)	32.3 (15.1)	17.4 (−0.2 to 37.2)
**Hallux**	72.3 (75.1)	50.9 (34.0)	41.9^a^ (2.9 to 100.0)	46.5 (19.0)	45.9 (18.5)	1.3 (−13.1 to 15.9)

*CI* confidence interval, ^a^significant at 0.05 level, Difference: calculated with participants < 2 ft pathologies as base group

For Toe PTI for < 2 ft biomechanical pathologies, subject 119 was an outlier and was removed from calculations

## Discussion

The results of this study show that simple clinical measures of foot biomechanical function are associated with elevated plantar pressure variables in a group of community dwelling adults with diabetes. Despite being what would be considered a predominantly low risk group, this cohort presented with mean barefoot (687.6 kPa) and in-shoe (234.1 kPa) forefoot peak pressure values that have been associated with increased risk of DFU development [29, 30]. While no exact plantar pressure cut-off value for ulceration has been identified, Lavery et al. [29] have shown that people with peak barefoot plantar pressures > 650 kPa are at a six times greater risk for ulceration than people with pressures below this value, and the IWGDF guidelines recommend an in-shoe peak pressure of < 200 kPa to reduce ulcer risk [10].

After grouping participants into those with two or more foot biomechanical pathologies and those with less, the group with two or more pathologies displayed higher barefoot peak pressures and pressure time integrals in all foot regions, although the forefoot increases did not reach statistical significance. Without costly and time-consuming plantar pressure testing equipment, the elevated plantar pressures present in this cohort would not be able to be detected during a standard podiatry consultation. No significant differences were seen between the two groups for in-shoe plantar pressure variables. This may be explained by the effects of the sports shoe used for in-shoe pressure measurement. Lower plantar pressure variables can be expected in-shoe compared to barefoot as the sports shoes used in this trial are designed to provide impact attenuation for the body by altering the ground reaction force and rate of loading [31]. This is consistent with previous studies in people with diabetes which revealed that the use of running shoes resulted in reductions in plantar pressures of 31 to 47% at the forefoot and 32% at the hallux compared to Oxford shoes [32, 33].

Not surprisingly, the individual biomechanical foot pathologies were seen to exert their effects in different foot regions, although some unexpected outcomes were seen. Contrary to previous studies, ankle dorsiflexion ROM was not a significant determinant of forefoot pressure variables but was significant for the lesser toes and hallux, both barefoot and in-shoe. In people with diabetes, a non weight bearing ankle dorsiflexion restriction has been shown to result in higher prolonged weight bearing at the forefoot and increased plantar pressures [34–36]. The difference in results may be partly explained by the fact that the previous studies measured ankle dorsiflexion in a non-weight bearing position compared to weight bearing in this study. It may also be the case that only more substantial ankle dorsiflexion restrictions, such as a weight bearing equinus restriction where ankle dorsiflexion < 30 degrees, result in gait alterations that affect forefoot plantar pressures [21].

The presence of HAV and lesser toe deformities only affected barefoot pressure variables. HAV resulted in significantly higher PP at the forefoot, toes and hallux along and an increased midfoot PTI. This may be because the first metatarsophalangeal joint functions as a pivot during the propulsion stage of gait, and HAV renders this mechanism less effective, resulting in altered forefoot loading [37]. It was unexpected that the presence of lesser toe deformities did not result in local lesser toe plantar pressure increases, which is where inspection for effects is most commonly suggested [38]. Instead, the presence of toe deformities had the effect of increasing rearfoot and forefoot PP, and rearfoot, midfoot and hallux PTIs. Claw and hammer toes contribute to a plantarflexed metatarsal which could explain the increased peak forefoot pressure [39]. The increased loading in the mid and rearfoot are possibly gait compensations to offload pain at the toe and metatarsal secondary to the toe deformity and therefore this may not translate across neuropathic cohorts [40]. The plantar cushioning and adequate toe box space provided by the sports shoe may explain why these factors did not affect in-shoe pressure variables.

Hallux range of motion was also a significant variable affecting plantar pressures. It had the effect of significantly increasing in-shoe forefoot peak pressures and PTIs, and significantly decreasing barefoot hallux and lesser toe peak pressures and hallux PTIs, and in-shoe hallux peak pressures and PTIs. Increased hallux ROM resulted in elevated pressure at the forefoot possibly due to first ray plantarflexion during normal propulsive phase of gait, with a corresponding reduction in pressure at the hallux [41]. Further, reduced hallux ROM (hallux limitus) demonstrated increased pressure at the hallux and lesser toes. A restriction at the hallux is thought to prevent normal propulsion through plantarflexion of the first ray leading to propulsion through the lateral foot and a consequent increase in pressure at the lateral toes as well as the hallux due to hyperextension of the hallux interphalangeal joint [41]. These findings are consistent with those reported in previous studies [27, 42].

Of the two non-biomechanical factors examined, BMI was seen to exert more wide-ranging effects than neuropathy. BMI had a significant effect on both barefoot and in-shoe midfoot PPs and PTIs, in-shoe forefoot PP and PTI, barefoot toe, forefoot and rearfoot PTIs and in-shoe toe PTIs. This agrees with prior studies that show a high body mass and high BMI adversely affect plantar pressures [17, 43, 44]. In this study the only significant effect that neuropathy had was on in-shoe forefoot PTIs. Neuropathy has previously been associated with increased plantar pressure variables [18] and also with the development of the majority of foot ulcers [45], so it may be the case that the neuropathy seen in this community dwelling group may not yet be severe enough to adversely impact PP variables.

The particular foot pathologies that affected plantar pressure regions differed between barefoot and in-shoe conditions. This was not unexpected as a 2015 systematic review by Franklin et al. [46] has reported a change in gait parameters when barefoot in comparison to in-shoe. These include a reduced step and/or stride length, increased ankle plantarflexion, as well as decreased stance time and decreased double support time when barefoot compared to in-shoe [46]. In addition, a reduced initial vertical impact force and more even distribution of pressure across the foot is experienced when walking barefoot which is likely to be as a result of a larger contact surface area achieved via a flatter foot placement [46]. While these altered kinematic and kinetic effects are plausible influences to explain the different predictors of pressure between the barefoot and in-shoe condition, the properties of the shoe may also be important. In addition to a soft cushion sole, the standardised shoe used (New Balance® 624) has a small bevel to the anterior and posterior sole of the shoe, effectively making a rocker sole, which has been shown to reduce forefoot plantar pressures and restrict sagittal plane range of motion [47].

There are calls for greater efforts to delay or prevent the first DFU in people with diabetes [48]. While high plantar pressures have been associated both retrospectively and prospectively with increased DFU risk [11–13] they can be easily overlooked by primary care clinicians as they present without obvious clinical signs and symptoms. Our results show that fast, low cost clinical tests may be used to assist with early detection of elevated plantar pressures in people with diabetes. The tests may be appropriate in situations where plantar pressure testing equipment is not available, or when screening low risk diabetes participants where costly testing or further referral is not warranted. A number of first line options are available to primary care clinicians who suspect high plantar pressures in their clients with diabetes. As shown in this and other trials, walking barefoot is associated with higher plantar pressures in older people both with and without diabetes, and a recommendation to wear relatively inexpensive off the shelf running shoes can help to alleviate high plantar pressures [33, 49]. Other footwear such as rocker bottom shoes have also shown peak pressure reductions of approximately 50% in the central forefoot compared to Oxford and semi-orthopaedic shoes in people with diabetes [50]. Patient education, regularly scheduled foot examinations and timely treatment of pre ulcerative signs (callus, blisters, fungal infections and thickened or ingrown toenails) may also be considered by the clinician who suspects the presence of high plantar pressures [48].

The results of this study should be considered in light of several limitations. Only a selected number of easily measured clinical variables were used in the multivariate multiple linear regression analysis. Previous studies in non-diabetes populations have identified other factors, such as measurement of foot and ankle structure derived from radiographs and muscle activity [51, 52] that impact plantar pressures, and the variance not accounted for in this model may be due to the omission of further predictor variables. Similarly we did not sub-classify toe deformities into reducible and non-reducible deformities with the different deformity type potentially having differing impacts on plantar pressures. Additionally, the non-probability sampling method and lack of vascular assessment means it is difficult to determine how generalisable our results are to the general population with diabetes. However, the relatively low incidence of LOPs and the small number of participants affected by previous DFU suggest this may be a largely low risk cohort and we are likely to see more significant changes in plantar pressures in higher risk cohorts. Further research could confirm these results in higher risk diabetes cohorts and also investigate which combination of clinical tests are superior in detecting elevated plantar pressures.

## Conclusions

Easily measured foot deformities and joint limitations significantly contributed to elevated plantar pressure variables in a largely low risk group of community dwelling adults with diabetes. Participants presenting with two or more of these foot pathologies displayed higher peak pressures and pressure time integrals in all foot regions than those with less than two pathologies. Elevated plantar pressures have been associated in the literature with increased DFU risk, DFU recurrence and delayed DFU healing in people with diabetes. These tests may be used to allow early detection of this mostly asymptomatic DFU risk factor, especially where plantar pressure testing equipment is unavailable.

## Supplementary Information

**Additional file 1:.** Pedar (left) and HRMAT (right) foot mask example

**Additional file 2:.** Residual vs Fitted Values for the 10 plantar pressure variables

## Data Availability

The datasets used and/or analysed during the current study are available from the corresponding author on reasonable request.

## Abbreviations

BMI: Body mass index
CI: Confidence interval
DFU: Diabetes related foot ulcer
HAV: Hallux abductus valgus
LOPS: Loss of protective foot sensation
PP: Peak pressure
PTI: Pressure time integral
ROM: Range of motion
