# Supplementary figures and images for: Clinical foot measurements as a proxy for plantar pressure testing in people with diabetes
Source: J Foot Ankle Res. 2021 Oct 27;14:56. doi: 10.1186/s13047-021-00494-4 (PMC8549160; doi:10.1186/s13047-021-00494-4)

**Additional File 1: Pedar (left) and HRMAT (right) foot mask example**

| 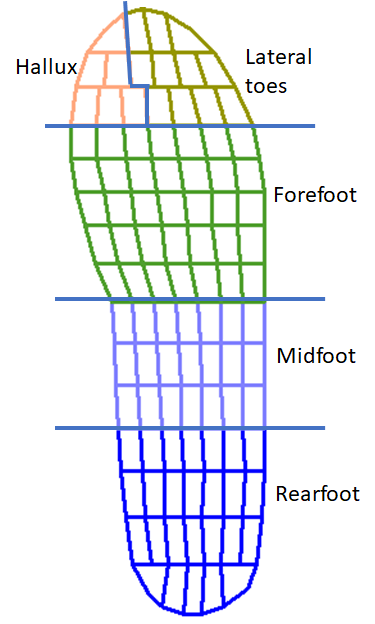 | 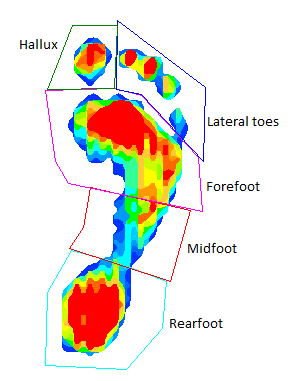 |
| --- | --- |

Supplement: Supplementary file 1 — Additional file 1:. Pedar (left) and HRMAT (right) foot mask example [file 13047_2021_494_MOESM1_ESM.docx]
