# Supplementary material for: Clinical foot measurements as a proxy for plantar pressure testing in people with diabetes
Source: J Foot Ankle Res. 2021 Oct 27;14:56. doi: 10.1186/s13047-021-00494-4 (PMC8549160; doi:10.1186/s13047-021-00494-4)
Supplement: Supplementary file 2 — Additional file 2:. Residual vs Fitted Values for the 10 plantar pressure variables [file 13047_2021_494_MOESM2_ESM.docx]

**Additional File 2: Residual vs Fitted Values for the 10 plantar pressure variables**

(A)

(B)

(C)

(D)

(E)

(F)

(G)

(H)

(I)

(J)
